# Ocean deoxygenation, the global phosphorus cycle and the possibility of human-caused large-scale ocean anoxia

**DOI:** 10.1098/rsta.2016.0318

**Published:** 2017-08-07

**Authors:** Andrew J. Watson, Timothy M. Lenton, Benjamin J. W. Mills

**Affiliations:** 1 Earth System Science Group, College of Life and Environmental Sciences, University of Exeter, Exeter EX4 4QE, UK; 2 School of Earth and Environment, University of Leeds, Leeds LS2 9JT, UK

**Keywords:** oceanic anoxic events, biogeochemical cycles, global change, phosphate, Redfield ratios

## Abstract

The major biogeochemical cycles that keep the present-day Earth habitable are linked by a network of feedbacks, which has led to a broadly stable chemical composition of the oceans and atmosphere over hundreds of millions of years. This includes the processes that control both the atmospheric and oceanic concentrations of oxygen. However, one notable exception to the generally well-behaved dynamics of this system is the propensity for episodes of ocean anoxia to occur and to persist for 10^5^–10^6^ years, these ocean anoxic events (OAEs) being particularly associated with warm ‘greenhouse’ climates. A powerful mechanism responsible for past OAEs was an increase in phosphorus supply to the oceans, leading to higher ocean productivity and oxygen demand in subsurface water. This can be amplified by positive feedbacks on the nutrient content of the ocean, with low oxygen promoting further release of phosphorus from ocean sediments, leading to a potentially self-sustaining condition of deoxygenation. We use a simple model for phosphorus in the ocean to explore this feedback, and to evaluate the potential for humans to bring on global-scale anoxia by enhancing P supply to the oceans. While this is not an immediate global change concern, it is a future possibility on millennial and longer time scales, when considering both phosphate rock mining and increased chemical weathering due to climate change. Ocean deoxygenation, once begun, may be self-sustaining and eventually could result in long-lasting and unpleasant consequences for the Earth's biosphere.

This article is part of the themed issue ‘Ocean ventilation and deoxygenation in a warming world’.

## 1. Introduction

The oxygen content of the ocean today is finely balanced. In the oxygen minimum zones (OMZs), to be found in the pycnocline of every ocean basin, O_2_ is depleted by respiring organisms feeding on sinking biological material originating from photosynthesis at the surface. In a few regions such as the eastern Pacific and the northern Indian Ocean, which are far from deep water formation regions and where surface productivity is high, the depletion is sufficient to completely or almost completely deoxygenate the water [1]. Over most of the ocean, the depletion is substantial but not complete.

Both models [2–5] and simple calculations (see below) suggest that a modest (e.g. approx. 2×) increase in the new production of the global ocean, or decrease by a similar factor in atmospheric oxygen, would be sufficient to drive much larger volumes of water towards anoxia. Once dissolved O_2_ falls to a small percentage of surface values, denitrification—bacterial reduction of nitrate—begins to occur, as is seen today in a number of OMZs such as that of the Arabian Sea [6]. When the small reservoir of nitrate is exhausted, microbial reduction in sulfate may begin and the waters become euxinic, with production of hydrogen sulfide. Though there are no regions of the open ocean today that exhibit permanently euxinic conditions, they are found in the Black Sea below 100 m, and in restricted basins such as some fjords, where most of the sulfate reduction actually occurs in underlying sediments rather than the water column. There is good evidence for much more widespread euxinic conditions at intervals in the past [7].

As first pointed out by Redfield [8], a simple calculation implies a close balance between nutrients and oxygen in the oceans. Table 1 summarizes Redfield's argument, updated to use more recent values of stoichiometric ratios. We start from the average deep ocean concentration of phosphate (which we take to be the ultimate limiting nutrient [10]) in the global ocean, of 2.2 µmol kg^−1^ [11]. P is supplied to the surface ocean by the upwelling of deeper water. By mass conservation, an equivalent volume of water must be subducted from the surface mixed layer into the interior, carrying oxygen dissolved from the atmosphere, such that the supply of O_2_ to the interior ocean is set by the solubility of oxygen and the atmospheric concentration [1]. Redfield assumed that all the nutrients at the surface are used in photosynthesis to fix organic carbon, and this then sinks into the deep sea and is respired and remineralized, consuming the dissolved oxygen. He used the known concentrations of PO_4_ in deep water and the stoichiometric ratios, which now bear his name, to calculate the oxygen demand. We use the ‘best guess’ value given by Anderson [9] of –O_2_/P = 150. The computed oxygen demand and the oxygen supply are almost equal to one another, suggesting that essentially all the oxygen carried into the interior of the ocean would be used in respiration.

**Table 1.** Representative calculation of the approximate oxygen demand per litre of upwelled water, and oxygen supply per litre of subducted water. Concentrations are in µmol kg^−1^.

PO_4_ concentration in deep water	carbon fixed (= 106 × PO_4_)	O_2_ demand (150 × PO_4_ [9])	saturated [O_2_] in equilibrium with atmosphere at 5°C and *S* = 35
2.2	106 × 2.2 = 233	2.2 × 150 = 330	320

If the assumptions of this calculation were correct, it would imply that the entire deep ocean today should be hypoxic, but it is not. Oxygen demand is set not only by the concentration of the limiting nutrient phosphate in the deep ocean, but also by the efficiency with which the nutrient is used by the biota when the water is upwelled to the surface. The Redfield calculation of table 1 assumes that this efficiency is 100%, but, globally averaged, it is now believed that only 30–40% of the phosphate in the deep ocean has arrived there via the biological pump: the remainder is ‘preformed’, meaning that it was present in the water, unused by the biota, when it subducted from the surface [12]. Thus, the calculation overestimates oxygen demand by a factor of two or three, and most of the deep sea is well oxygenated, with hypoxia restricted to regions far from deep water formation sites, and underlying zones of high productivity. Nevertheless, models and simple calculations are in agreement that the ocean biogeochemical system responds such that small changes in oxygenation or net primary production substantially affect the volume of hypoxic water, and the area of sediments overlain by hypoxic waters. In this sense, the ocean could be described as being on the ‘edge of anoxia’ [13]. Is there a deeper reason why the ocean, globally, should behave in this way, or is this just chance?

**Figure 1.**
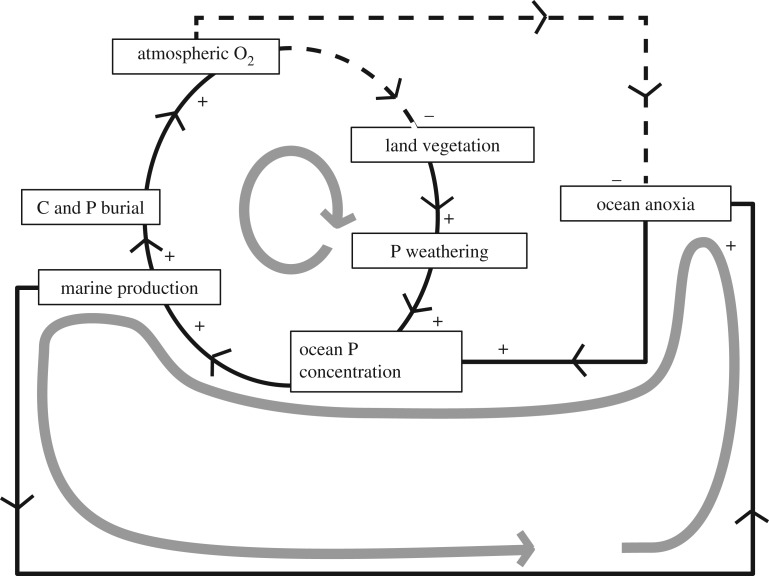
Symbol and arrow representation of some Earth system interactions linking atmospheric oxygen and ocean nutrients. Direct relations are shown by solid lines with a plus sign, and inverse relations shown by dashed lines and a minus sign—so, for example, an increase in C and P burial causes an increase in atmospheric O_2_, but an increase in atmospheric O_2_ causes a decrease in land vegetation. Where arrows describe a closed circuit, this indicates a feedback loop, which is negative, i.e. stabilizing, where there is one dashed arrow in the loop, and positive if there are none. Thick grey lines highlight two feedback loops. Upper loop: a negative feedback stabilizes atmospheric O_2_ and ocean P concentrations via the influence of the atmosphere on land vegetation (principally by wildfire), land vegetation influence on marine P via continental weathering and marine P influence on atmospheric O_2_ by stimulating marine production and carbon burial. Lower loop: positive feedback that may be important in sustaining OAEs. Ocean anoxia releases P from sediments, thus increasing P concentrations, enhancing marine production, which further increases anoxia.

We suspect it is not chance, but is ultimately dictated by the network of feedbacks which link the phosphorus cycle with the redox state of the ocean and atmosphere. These feedbacks are important for the regulation of atmospheric oxygen concentration at levels not too far from that of the present day, concentrations that have been maintained for the past several hundred million years [13–15, 16, 17]. Figure 1 illustrates in symbol-and-arrow form a number of possible feedbacks that link atmospheric and ocean oxygen concentrations and ocean nutrients. On geological time scales, the source of bio-available phosphorus to the oceans is the weathering of continental rocks, with the products delivered via rivers. This weathering is strongly accelerated by the terrestrial biota, with land plants, animals, fungi and the soil microflora all playing important roles [18]. Trees and forests are particularly important, because vascular plants with extensive root systems promote weathering of rock, soil formation and bio-availability of phosphorus, though even the earliest land plants are thought to have accelerated P weathering [19–22]. Maintaining relatively stable concentrations over million-year time scales requires the source of phosphorus from continental weathering to balance the sink flux out of the oceans, which is burial in marine sediments. Phosphorus is transported to the sediments alongside organic carbon [23], and this burial of organic carbon is the ultimate source of free oxygen in the atmosphere [14,24]. The rise of land plants therefore forced up atmospheric oxygen by accelerating carbon burial at the same time as it increased phosphorus weathering. Land plants are, however, limited by atmospheric O_2_ concentrations: in particular, if O_2_ rises too high, there is an increased prevalence of wildfire, which then reduces tree and forest cover, and therefore the extent to which land plants accelerate P weathering. Models of this network of feedbacks [14] suggest that, at steady state, oxygen concentrations are controlled in a ‘fire window’ similar to present levels, while marine phosphate concentrations adjust, so that the oceans are not far from the Redfield stoichiometric ratio with respect to oxygen.

Though the original Redfield calculation overstates the closeness of the balance between biological oxygen demand and O_2_ supply, the size and intensity of the major deoxygenated zones are still sensitive to quite small changes in ocean physics or geochemistry. Thus, many models suggest that, if the concentration of phosphate in the deep sea were to increase significantly (or, equivalently, if changes in ocean circulation were to increase the efficiency of the existing biological utilization of PO_4_), a substantially greater volume of ocean water would be depleted in oxygen [2–5,25]. This sensitivity of the low-oxygen regions to relatively small changes in circulation or oxygen supply may in part help to explain observed changes occurring today in the OMZs, under the influence of ongoing climate change [26]. It may also help to explain the frequent occurrence in the geological record of major ‘oceanic anoxic events' (OAEs) [27], to which we now turn.

## 2. Ocean anoxic events

While the deep sea today is well oxygenated, there is evidence for numerous OAEs in the past [27], especially in the Mesozoic Era. OAEs, first recognized in the 1970s [28], are identified by discrete layers of black shales having high organic carbon content, with coeval deposits often widely separated in space, indicating global as well as local causes. They are often accompanied by excursions in *δ*^13^C (both positive and negative in sign), which indicate major upheaval in the global carbon cycle, with the ocean anoxia implied being over substantial basins, but not global in extent [27]. Some, but by no means all, of these events accompany well-known environmental crisis points and mass extinctions, for example, the Permian–Triassic boundary [29]. OAEs typically last for periods of approximately 10^5^–10^6^ years.

OAEs seem to occur exclusively during past warm ‘greenhouse’ periods of Earth's climate, and the expansion of anoxia has been considered one of the defining features of extreme ‘hothouse’ episodes occurring within these warm greenhouse periods [30,31]. Several factors induced by warm climates might contribute to the spread of anoxia. Oxygen solubility decreases with increasing temperature [32], being about 30% less at 20°C than at 2°C, for example, so that less O_2_ will be dissolved in warmer ocean water [33]. Furthermore, warm climates, at least those induced by increased CO_2_ concentrations in the atmosphere such as during the Cretaceous, have smaller equator-to-pole differences in temperature, with the Jurassic and Cretaceous Earth in particular being free of icecaps. To the extent that density differences between warmer and colder waters enhance the meridional overturning circulation, we might expect that such warm climates would have less vigorous deep water formation, and oceans therefore less well ventilated, than cold periods [33]. (However, meridional density differences are only one determinant of the overturning circulation [34].)

A more compelling reason why OAEs may be associated with warm periods, and especially hothouse episodes within them, is that a global warming event, especially if abruptly triggered, is expected to increase the rate of weathering of continental rocks—through both increased humidity and runoff, and the direct effect of temperature on reaction kinetics. Increasing the supply of nutrients to the ocean thereby increases global marine productivity and oxygen demand in subsurface water. An increase in nutrient supply leading to eutrophication of the oceans is indicated by model studies to be the most likely cause for past OAEs [2,4,25,35] and is also indicated by interpretation of geological evidence [7,36]. It is more powerful in promoting low oxygen than the direct effects of temperature on solubility or circulation, because the effect of increased temperatures on solubility of oxygen is relatively modest, while one effect of reduction in ventilation is to decrease productivity because it implies a reduction in overturning, and hence of the upwelling of nutrient-rich water to the surface.

The massive volcanic and magmatic events that emplace large igneous provinces (LIPs) have now been implicated in the genesis of several OAEs [30,31,37,38]. LIPs are expected to increase the supply of nutrients to the oceans, not only because they might trigger a global warming episode as a result of CO_2_ and other greenhouse gases released to the atmosphere, but also because the large volumes of rapidly weathering basalt rocks emplaced by the eruption would be an extra source of phosphorus and potentially also trace-element nutrients.

## 3. Phosphorus cycling and ocean anoxia

As discussed above, we have cause to believe that there is a negative feedback involving atmospheric oxygen and the long-term source of phosphorus to the ocean, which ties these variables together and regulates them over multi-million-year time scales. Figure 1 also highlights a positive feedback between ocean anoxia and oceanic P concentrations, which may play a role in promoting OAEs on somewhat shorter time scales. The sink for marine phosphorus is incorporation in marine sediments, transported there mostly in organically derived detritus, though much of it is subsequently transformed to authigenic minerals [39,40]. It is well established that sediments in contact with oxygen-depleted water normally have lower concentrations of reactive P relative to organic carbon, with much of the P being regenerated to the water column under anoxic conditions [41–43]. The P thus released back into the water can act to further boost marine productivity, leading to increased eutrophication and more anoxia. This mechanism is well established to occur in the largest modern coastal hypoxic zone, the Baltic Sea, where the variation in phosphate content of the low-oxygen water indicates that most of it is regenerated from low-oxygen sediments rather than input from the land [44]. It is also believed to be significant in the second largest such zone, in the Gulf of Mexico [45]. It would be expected that this mechanism would also be important for the global ocean in generating and sustaining past OAEs, and models suggest this is the case [2,4,25,35].

The time scale for onset of eutrophication-driven global anoxia will be set by the residence time of bio-available P in the global ocean. Even for the modern ocean, there is uncertainty about this number, mirroring the complexity of the phosphorus cycle, but it probably lies in the range (15–35) × 10^4^ years [23,46]. Major changes in the total ocean concentration of P are therefore unlikely to occur on time scales shorter than thousands of years, so we may conclude that a global-scale nutrient-induced OAE is not an immediate global change concern. It is nevertheless of interest to explore in a semi-quantitative way the consequences for marine oxygen of our changing the phosphorus cycle on these longer time scales. Specifically, we can ask (i) how fast might oxygen concentrations decrease as a result of human-caused acceleration of the P cycle, (ii) what role might the positive feedback on P concentrations play, and (iii) whether known phosphatic rock reserves are sufficient that, if all were fully used, major ocean deoxygenation would occur? As an aid to this, we introduce a simple model which captures some aspects of the positive feedback on ocean anoxia in a semi-quantitative manner. Our model is not spatially resolved, unlike those of Palastanga *et al*. [3] or Niemeyer *et al*. [47], both of whom have recently published studies using three-dimensional and intermediate complexity models of the ocean biogeochemistry, focusing on the long-term implications of anthropogenic change on the P cycle. However, P recycling is likely to be very nonlinear, dependent in particular on the degree to which low-oxygen waters encroach onto the continental shelves and slopes where the organic and P-rich sediments are laid down. To be accurately represented, this requires high spatial resolution, but to date the spatial resolution of the models employed is quite coarse. Our model represents this nonlinearity by an analytical function (described below), which, while obviously an oversimplification, is at least transparent in its operation and readily manipulated.

## 4. Model description

The model we describe here is inspired by earlier published studies [25,48,49]. The master variable is the ocean P reservoir, denoted *P* the rate of change of which is given by

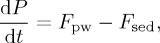

where *F*_pw_ is the input of phosphorus from weathering and *F*_sed_ is the flux that is lost to sediments. The sedimentation term is

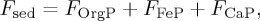

where the three terms are the forms of P removal to sediments that are commonly modelled: *F*_OrgP_ is burial associated with organic matter, *F*_FeP_ the burial adsorbed to iron oxides and *F*_CaP_ the burial associated with authigenic carbonate minerals. For (pre-industrial) present-day fluxes (in GmolP yr^−1^), we follow Slomp & Van Cappellen [49] with *F*_pw_ = 92, *F*_OrgP_ = 23, *F*_FeP_ = *k*_FeP_ = 23 and *F*_CaP_ = *k*_CaP_ = 46. For a present reservoir of *P*_0_ = 3.1 × 10^15^ mol, this gives an oceanic residence time of P of approximately 34 kyr. We vary the input of phosphorus to the ocean with a weathering control parameter, *W*:

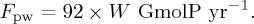

So *W* = 1 corresponds to the Slomp & Van Cappellen [49] steady state. The supply of phosphorus and organic carbon to the sediments in sinking organic matter is assumed to be proportional to the P reservoir size (i.e. [PO_4_]), which governs export production (assuming fixed circulation). This phosphorus input to sediments can then be buried in its original form (Org-P), or subject to diagenesis, following which much of it can be incorporated into the sediment as authigenic carbonate minerals (Ca-P) and some by adsorption to iron oxides (Fe-P), or else it may be released back to the water column.

Organic phosphorus burial is sensitive to the fraction of the seafloor overlain by anoxic waters, *f*_anoxic_, given very different (C/P)_organic_ burial ratios under anoxic (CP_anoxic_) and oxic (CP_oxic_) bottom waters:

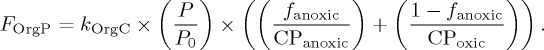

Here, we take CP_anoxic_ = 4000 and CP_oxic_ = 250 following earlier work [50]. Given a very small *f*_anoxic_ at present (see below), the overall (C/P)_organic_ burial ratio is approximately 250 and an organic carbon burial flux of *k*_OrgC_ = 5.75 TmolC yr^−1^ corresponds to *F*_OrgP_ = 23 GmolP yr^−1^.

Phosphorus is desorbed from the surface of iron oxides under anoxic bottom waters, hence iron-sorbed phosphorus burial scales with the oxic fraction of seafloor:

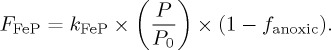


Calcium-bound phosphorus burial is assumed to simply scale with the P input to sediments from sinking organic matter, although other work considers a redox sensitivity of this flux [49]:

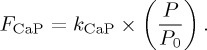


The fraction of seafloor overlain by anoxic waters, *f*_anoxic_, is assumed to follow a logistic functional form dependent on the balance between normalized oxygen demand *k*_U_ × (*P*/*P*_0_) and oxygen supply (O_2_/O_20_):

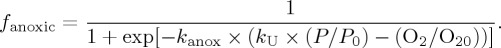

Here *k*_U_ represents the (in)efficiency of nutrient utilization in upwelling regions. We choose *k*_U_ = 0.4 based on the estimates of preformed nutrient efficiency [27] and also agreement across a range of models that a transition from an oxic to an anoxic deep ocean occurs at O_2_/O_20_ approximately 0.4 for present nutrient levels, or at *P*/*P*_0_ approximately 2.5 for present O_2_ levels [51–54]. The value *k*_anox_ = 10 is chosen based on observations that approximately 0.2–0.3% of the seafloor is overlain by anoxic bottom waters at present [55], and agreement among models that the transition from oxic to anoxic conditions is abrupt, occurring typically as O_2_/O_20_ drops from approximately 0.5 to approximately 0.3 [51,53,54]. Here, we treat atmospheric O_2_ as a constant, i.e. O_2_/O_20_ = 1, because it varies on a much longer time scale than ocean P and we wish to focus on the conditions required to trigger ocean anoxia.

Such a simple model of the anoxic fraction fails to capture the effects of ocean hypsometry and in particular the disproportionate contribution of shallow shelf seas to phosphorus removal and recycling. When anoxia impinges on the bottom of the shelf seas, we may expect a stronger positive feedback from benthic phosphorus recycling, as seen in the results of Ozaki and co-workers [54]. Such a nonlinearity is hard to capture in coarse-resolution global ocean models, including GENIE and HAMOCC, because they barely resolve shelf seas and their particular dynamics.

## 5. Results

**Figure 2.**
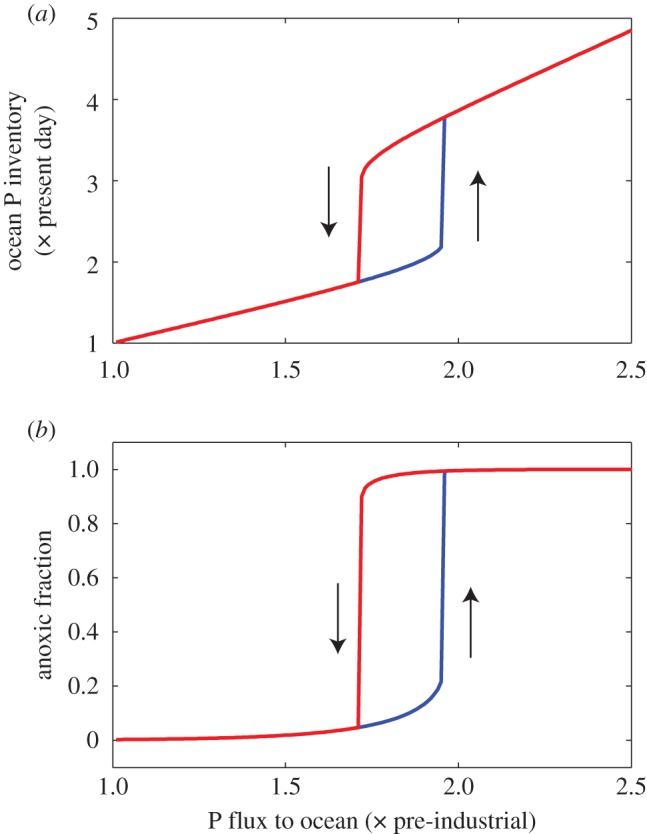
Steady-state solutions for oceanic P inventory and ‘anoxic fraction’ (fraction of ocean sediment overlain by anoxic water), for the simple model described in the text, as a function of the flux of P to the ocean. Arrows show the directions in which two stable solutions are found. (Online version in colour.)

The simple model outlined above encapsulates a strong sensitivity of ocean anoxia (*f*_anoxic_) to phosphorus input to the ocean, controlled by the weathering parameter *W*, where *W* = 1 represents the pre-industrial input. Figure 2 shows the behaviour of the steady-state solutions as a function of P input. As P input and oxygen consumption increase, expanding anoxia increases P regeneration from sediments until a tipping point is reached, beyond which the ocean abruptly shifts to a largely anoxic state. Decreasing the P input, the steady states eventually shift back to a largely oxic state, but this occurs at a different and lower P input. These dynamics result in a region of bistability, where either steady state is possible for a given value of *W*. For our choice of parameters, this bistable region is between about *W* = 1.75 and *W* = 1.95. In this region, therefore, a full OAE, once established, is sustained by the additional positive feedback effect on the P cycle. According to the model, the present-day increase of order 2 times in the P input to the ocean due to anthropogenic factors [56] would eventually cause such a shift if it were to be kept up for long enough.

In terms of the P content of the ocean, as *W* is increased and the tipping point is reached, ocean P is roughly doubled. Either side of the region of bistability, ocean P responds approximately linearly to P input (*W*), but the constant of proportionality increases from the oxic to the anoxic ocean solution because the (C/P)_organic_ burial ratio switches from 250 to 4000 and the Fe-P burial flux is removed.

**Figure 3.**
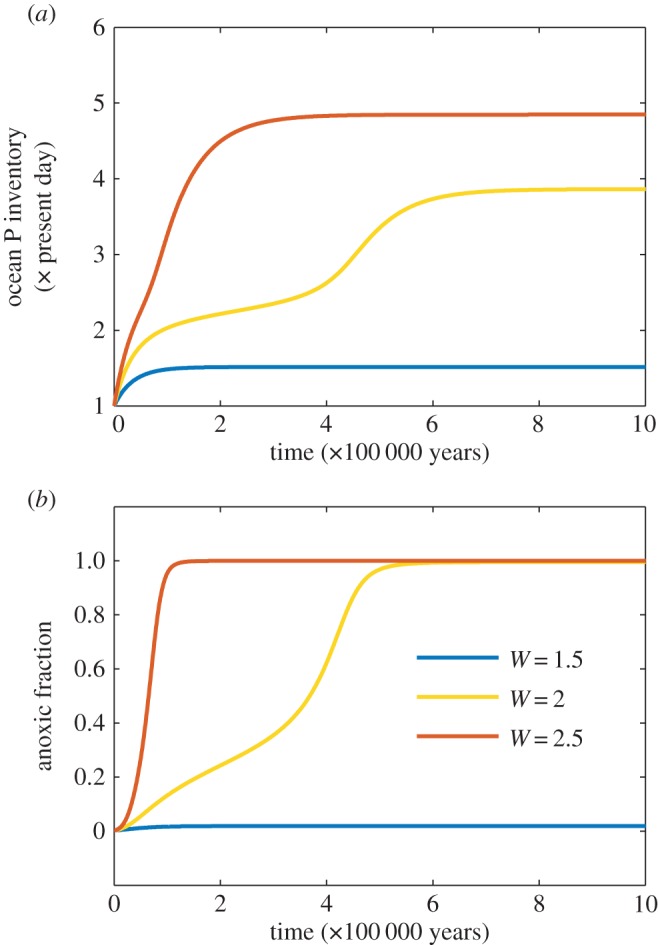
Evolution of modelled oceanic P inventory and anoxic fraction with time, for constant inputs of P to the oceans of 1.5, 2 and 2.5× the pre-industrial steady state.

Figure 3 shows transient runs starting from the pre-industrial steady state, with constant increased inputs of P: for *W* = 2.5, the transition to a fully anoxic ocean is established in a period of about 10^5^ years. For a value of *W* = 2, close to the tipping point, the transition occurs only after a long, quasi-static intermediate period, while for lower values, it does not occur.

**Figure 4.**
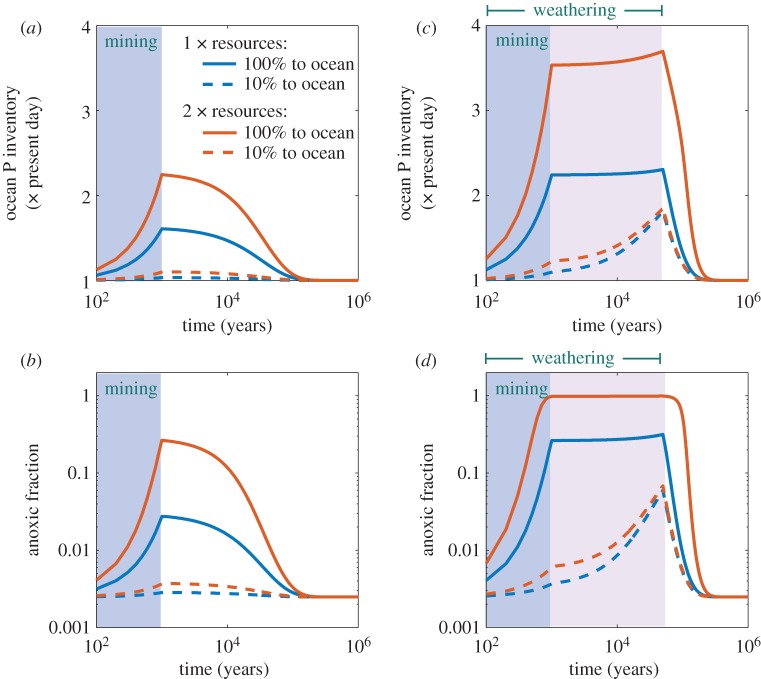
Evolution of modelled P inventory (*a*,*c*) and anoxic fraction (*b*,*d*) for future scenarios of enhanced P release into the oceans. The model runs are (*a*,*b*) release at a uniform rate over 1 kyr of multiples of the minimum phosphate-bearing rock resource (the known amount that might be extracted in the future, approx. 2 × 10^15^ mol P) as estimated by USGS [57]. Blue: 1× the resource; brown: 2× the resource; solid lines show 100% delivered to the ocean, dashed lines show 10% delivered to the ocean. (*c*,*d*) The four scenarios in *a*,*b* are combined with increased flux of P from 2 × enhanced weathering of continental rocks extending over 5 × 10^4^ years.

We can use the model to give semi-quantitative answers to questions about anthropogenic influence on global ocean deoxygenation via the phosphorus cycle:

(1) *Could the mining of phosphate ore materially influence global ocean anoxia?* The USGS estimates worldwide known reserves of phosphate rock, which generally has a P content of order 20%, as 69 Pg [57], suggesting a content of about 4.5 × 10^14^ mol P. This is about 15% of the present ocean inventory. Were we to mine all this phosphorus and in due course it were to be washed to the ocean, this is certainly enough to increase the prevalence of anoxia worldwide (there is, however, controversy over the USGS estimate of P reserves, with some arguing that it overstates the amount of known and proven ores [58]).
  However, worldwide *resources* (a broader classification than reserves, covering known phosphorus-containing deposits that might be exploited in the future) are estimated by the USGS at greater than 300 Pg rock, or perhaps 2 × 10^15^ mol P. If humanity were desperate enough for phosphorus in the future, additional resources may be discovered, while poorer-quality source rock with lesser P content might eventually be exploited. Such a quantity of P would, if it were all to end in the ocean in a bio-available form, have a very substantial effect on global ocean anoxia—in our model, releasing 2 × 10^15^ mol P over 1000 years causes the fraction of ocean sediment overlain by anoxic waters (*f*_anoxic_), to increase more than 10-fold, from 0.25% to peak at 2.7% (figure 4*a*,*b*, ‘1 × resources’ curve). Releasing twice this quantity causes the anoxic fraction to increase to approximately 20%. These scenarios represent an upper bound on the potential future delivery of phosphorus to the ocean. Projections by Filippelli [59] suggest total inputs may eventually be an order of magnitude lower than the mining input: phosphorus undergoes complex processes in the environment, and fertilizer phosphate may be retained for substantial periods in soils, river and lake sediments. Our upper bound assumes that this accumulated phosphorus will eventually be released to the ocean, such that a steady state is reached wherein terrestrial inputs and outputs balance [60]. A more conservative scenario is also shown in figure 4 (dashed lines) in which terrestrial P accumulation continues indefinitely and only 10% of the mining resource enters the ocean, in which case the effect on anoxia is quite minor.
(2) *Is it feasible that humans might bring about a full-scale ocean anoxic event?* From the above discussion, we can conclude that mining of currently known phosphate rock could have a huge impact on ocean anoxia, but would fall short of bringing on a full-scale OAE. However, just as past OAEs are thought to have been caused by increased weathering rates due to global warming events, human-caused warming might have a large additional impact on the oceans by increasing rates of nutrients released by rock and soil weathering. Anthropogenic warming from the spike of fossil-fuel-released CO_2_, which we are currently creating, will probably be appreciable for at least 10 000 years and perhaps up to 50 000 years into the future, as a result of the slow time scales on which CO_2_ is ultimately removed from the ocean–atmosphere system [61]. These periods are sufficiently long that comparatively modest increases in phosphate rock weathering due to the warming would have a substantial effect on the ocean inventory.

Figure 4*c*,*d* shows model results for an increase in P from weathering of soils and rocks due to global warming by a factor of two lasting 50 000 years, and this weathering source combined with the two mining scenarios. All these possible futures predict orders-of-magnitude enhancement of the fraction of the global ocean affected by anoxia. The most severe scenario tips the ocean into a full-scale OAE within 2000 years, from which it does not recover for nearly 100 000 years. Our results appear broadly consistent with those of Palastanga *et al.* [3] and other models, e.g. [2]. Palastanga *et al.* [3] do not show any model runs where whole-ocean anoxia occurs, but they do not consider the large factor of increase in P fluxes that our ‘mining’ scenarios imply. Such a large increase might cause anoxic waters to impinge on the sediments of shelf seas where most sedimentary P is contained, and could conceivably therefore trigger a nonlinear switch to global ocean anoxia as seen in our model and others [2].

## 6. Conclusion

It is generally believed that, to the extent to which deoxygenation observed to be occurring in the modern ocean is linked with anthropogenic global change, it is probably due to the direct effects of warming in slowing the ventilation of deep waters and decreasing the solubility of O_2_ in surface waters. The long-term effect of these mechanisms has been modelled by Shaffer *et al*. [62] and found to be considerable. However, for the case of the major ocean anoxic events in the past, which are often associated with warming episodes, the most powerful causal mechanism does not seem to have been these effects, but has been due to the global input of nutrient into the oceans [2,3,47]. Our very simple model suggests that, under unrestricted mobilization of P, this could be important in the future as a result of human activity. Both the deliberate mobilization of phosphorus for agriculture, and the incidental effects of global warming and anthropogenic increases in erosion rates in accelerating the natural weathering of phosphorus, could be important. Conceivably, if our descendants are heedless of the consequences and generate major increases in the flux of phosphorus to the oceans lasting for hundreds or thousands of years, large-scale and long-lasting global anoxia might result.

We might assume that the scenarios modelled here are more extreme than will ever be realized, even if our industrial civilization lasts thousands of years, because our increasing understanding of the Earth system will enable us to foresee the consequences: such sustained mobilization of P would be seen to be irresponsible and dangerous to the health of the whole Earth system, and action would be taken to ensure it did not occur. Let us hope we, and our descendants, are wise enough to take that course.

## Data accessibility

The code of the model described in the text and used to generate figures 2, 3 and 4 is written in MATLAB, and may be obtained from the authors.
